# Biological Roles and Clinical Significance of Exosome-Derived Noncoding RNAs in Bladder Cancer

**DOI:** 10.3389/fonc.2021.704703

**Published:** 2021-10-07

**Authors:** Yonghua Tong, Xiao Liu, Ding Xia, Ejun Peng, Xiaoqi Yang, Hailang Liu, Tao Ye, Xinguang Wang, Yu He, Hua Xu, Zhangqun Ye, Zhiqiang Chen, Kun Tang

**Affiliations:** Department of Urology, Tongji Hospital, Tongji Medical College, Huazhong University of Science and Technology, Wuhan, China

**Keywords:** exosome, non-coding RNA, bladder cancer, cancer biomarker, cancer therapy

## Abstract

Bladder cancer (BCa) is a common heterogeneous urinary system tumor with high malignancy and limited advancement in treatment. Limited understanding of BCa has not contributed to any significant progress in diagnosis or treatment, exploring the mechanisms underlying BCa has become an urgent research focus. Exosomes, a type of extracellular vesicle (EV), have drawn substantial interest for their important roles in mediating intracellular communication. Exosomes shuttle numerous bioactive molecules, and noncoding RNAs (ncRNAs) are among the most numerous. ncRNAs including microRNA, long noncoding RNA, and circular RNA are sorted and packaged into exosomes selectively and transferred into recipient cells to regulate their function. Exosomal ncRNAs are associated with hallmarks of BCa, such as proliferation, apoptosis, epithelial-mesenchymal transition (EMT), cell cycle arrest, lymphangiogenesis, and chemotherapy resistance. Exosomal ncRNAs can also be detected in urine and serum, making them encouraging biomarkers for BCa diagnosis and prognosis. More importantly, exosomes exhibit excellent biocompatibility and potential for diversified applications. The delivery of bioactive substances and drugs into specific cells has become a promising approach for precision therapy for BCa patients. In addition, cancer vaccines have also received increasing attention. In this review, we summarize the current research on the regulatory roles of exosomal ncRNAs in BCa tumorigenesis and progression, as well as their potential clinical value in accelerating the diagnosis and therapy of BCa.

## Introduction

Bladder cancer (BCa) is a urinary system tumor with high incidence and mortality, which is more common in elderly men (1). Based on the latest USA cancer statistics, BCa is still the fourth most common male malignancy, leading to an estimated 64,280 (7%) new cases and 12,260 (4%) deaths in 2021 (2). According to the depth of tumor infiltration into the bladder, BCa can be divided into two categories: nonmuscle-invasive bladder cancer (NMIBC) and muscle-invasive bladder cancer (MIBC) (3, 4). Nearly 90% cases of BCa are of the urothelial type, of which 20% are MIBC (5), and result in poor prognosis. Since the 1990s, clinicians have offered BCa patients the same, limited therapeutic options, including transurethral resection of bladder tumor (TURBT), chemotherapy, radiotherapy, and immunotherapy for NMIBC, and cystectomy as well as trimodality therapy for MIBC (1). Due to the lack of significant progress in treatment, the survival rates of BCa have not made any significant improvement over the last three decades, and approximately 60%–70% of NMIBC patients will experience disease recurrence even after surgery (5). Early diagnosis and treatment can help improve the long-term survival of BCa. At present, the diagnosis and follow-up of BCa mainly relies on cystoscopy, which is an invasive, costly, and unpleasant examination (1, 6). Therefore, elucidating the underlying molecular mechanisms of BCa carcinogenesis and progression and the identification of reliable and effective biomarkers for BCa diagnosis and monitoring have a crucial research focus.

BCa is a heterogeneous solid tumor consisting of more than 40 histological subgroups (7). Molecular technologies, such as DNA-sequencing and RNA profiling, have contributed to the definition of distinct biological subtypes of BCa, which are potentially useful for diagnosis, prognosis, and treatment (8). Numerous RNAs, especially noncoding RNAs (ncRNAs), have been found to be widespread in tissues and are cell type-specific, tissue-specific, or developmental stage-specific. ncRNAs are associated with BCa carcinogenesis and progression and participate in the regulation of BCa cell proliferation, epithelial-mesenchymal transition (EMT) processes, apoptosis, cell cycle arrest, treatment resistance, and the immunosuppressive microenvironment (9). Recently, exosomes, a type of extracellular vesicle (EV) carrying abundant elements including ncRNAs, have attracted the interest of many researchers. Exosomes form a “bridge” between cells and may be engaged in tumorigenesis and tumor development by delivering the ncRNAs and other components (10). In this review, we summarized the current research on the roles of exosomal ncRNAs in BCa progression as well as their potential clinical significance.

## Biogenesis and Characteristics of Exosomes

**Figure 1:**
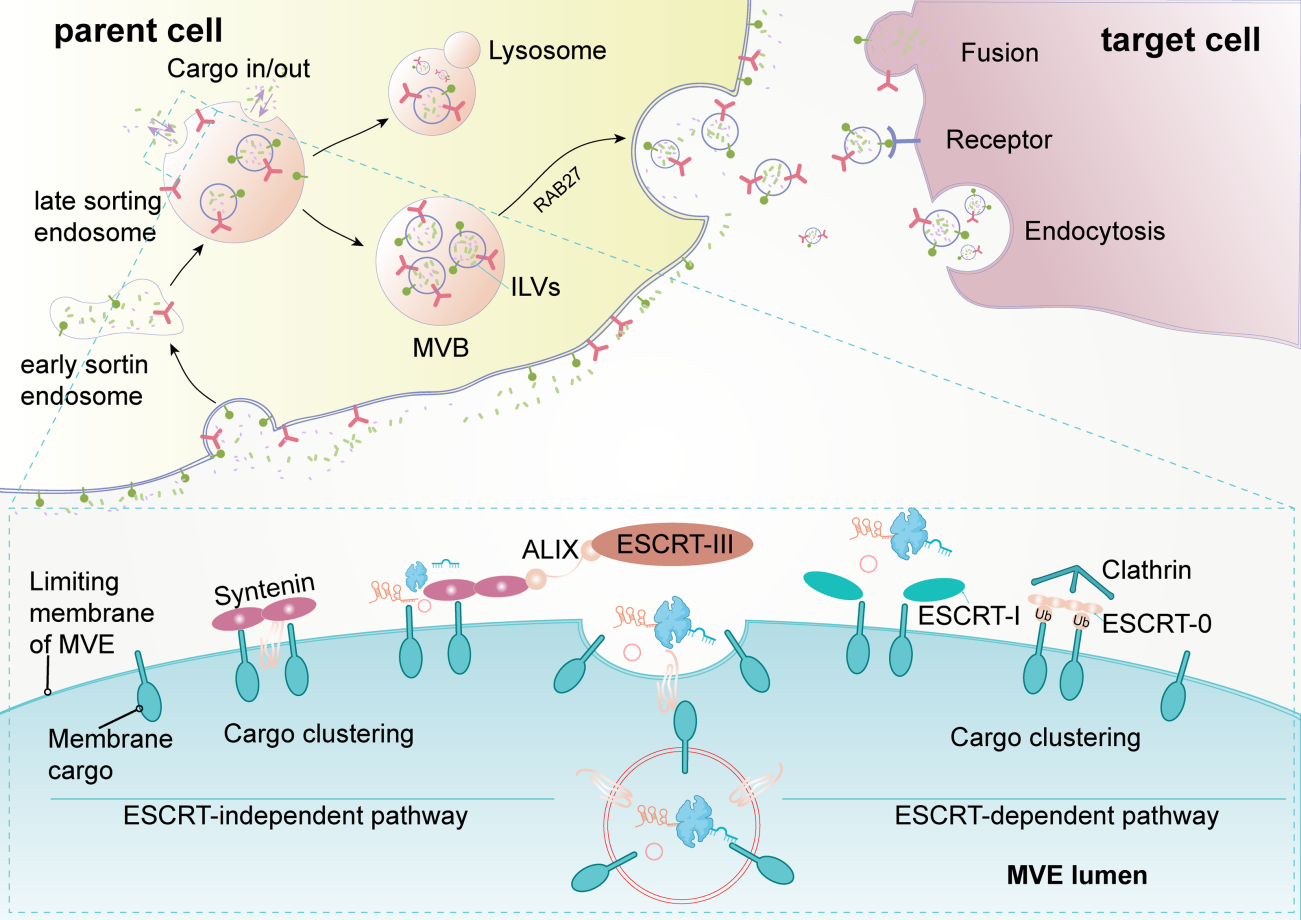
Biogenesis and release of exosomes (extracellular vesicles). The formation of exosomes is multistep, and the uptake of exosomes by recipient cells is also multimodal. Early sorting endosomes derived from bud formation at the plasma membrane maturate to multivesicular bodies (MVB) after the cargo sorting and exchange *via* an endosome-sorting complexes (ESCRT)-independent or ESCRT-dependent pathway. MVBs eventually directly fuse with lysosome and degradation or are transported to the plasma membrane for release. After secretion, recipient cells take up exosomes through fusion with the plasma membrane, receptor-mediated pathway, or endocytosis. ALIX, apoptosis-linked gene 2-interacting protein X; ILVs, intraluminal vesicles.

Exosomes are extracellular vesicles with a diameter of 30–150 nm that are enclosed by a lipid bilayer and are present in almost all body fluids (11, 12). Overall, the biogenesis and release of exosomes are complex multistep processes (**Figure 1**). Early endosomes are formed by the endocytosis of the plasma membrane. Then, early endosomes further mature into multivesicular bodies (MVB). Exosomes are generated as intraluminal vesicles (ILVs) during the maturation of MVBs, which are formed by inward budding of the endosomal membrane. In addition to fusion with the plasma membrane to secrete ILVs (future exosomes) into the extracellular environment, MVBs can also fuse with lysosomes for degradation. The endosome sorting complexes (ESCRT) required for the transport machinery play a critical role in the biogenesis of exosomes (12, 13). There are four types of ESCRT (ESCRT 0, ESCRT I, ESCRT II, and ESCRT III). ESCRT 0 and ESCRT I cluster specific cargoes and target them to the endosomal membrane, then the ESCRT I-ESCRT II complex initiates the inward budding process and recruits ESCRT III for ILV fission. Rab27A and Rab27B are required to transport MVB to specific membrane regions (14). In addition to ESCRT, some accessory proteins (ALIX, VPS4) are also involved in the regulation of exosome biogenesis (15). In addition, exosomes can continue to form after the depletion of the ESCRT complexes, indicating the presence of an ESCRT-independent pathway (16). After secretion, exosomes can naturally target adjacent or distant recipient cells in various ways (17).

Exosomes have previously been considered cellular dustbins for removing harmful substances, but recent studies have identified them as key mediators for cell-to-cell communication (18, 19). An increasing number of studies have reported that exosomes regulate both normal physiological activities in humans and the pathological conditions in many diseases. Exosomes contain a wide range of biological macromolecules, including proteins, nucleic acids, and lipids, which determine the exosomes’ functions and give them the ability to mediate many biological processes, including antigen presentation (20), wound healing (21), and tumor metastasis (22). Moreover, the potential clinical application of exosomes has also received increasing attention.

## Noncoding RNAs in Exosomes

With the advancement of research, exosomes were found to contain abundant molecular components (23). The contents of exosomes are diverse because these molecules are selectively sorted and MVB can fuse with other vesicles or organelles in the cell (24). Many studies have shown that exosomes contain a large number of different types of ncRNAs, with microRNA (miRNA) being the most representative, followed by long noncoding RNA (lncRNAs) and circRNAs, as well as a few PIWI-interacting RNAs (piRNAs) and tRNA-derived small RNAs (tsRNAs). These ncRNAs are involved in the regulation of several cell activities and cell-to-cell communication. The variety and quantity of ncRNAs contained in exosomes are influenced by the conditions of the parent cell as well as its surrounding microenvironment, and are transmitted to recipient cells to alter their destinies. Exosomal ncRNAs are expected to become biomarkers and potential therapeutic targets for diseases, especially tumors. The current review on BCa exosomes mainly focuses on miRNAs, lncRNAs, and circRNAs.

### MicroRNA

**Figure 2:**
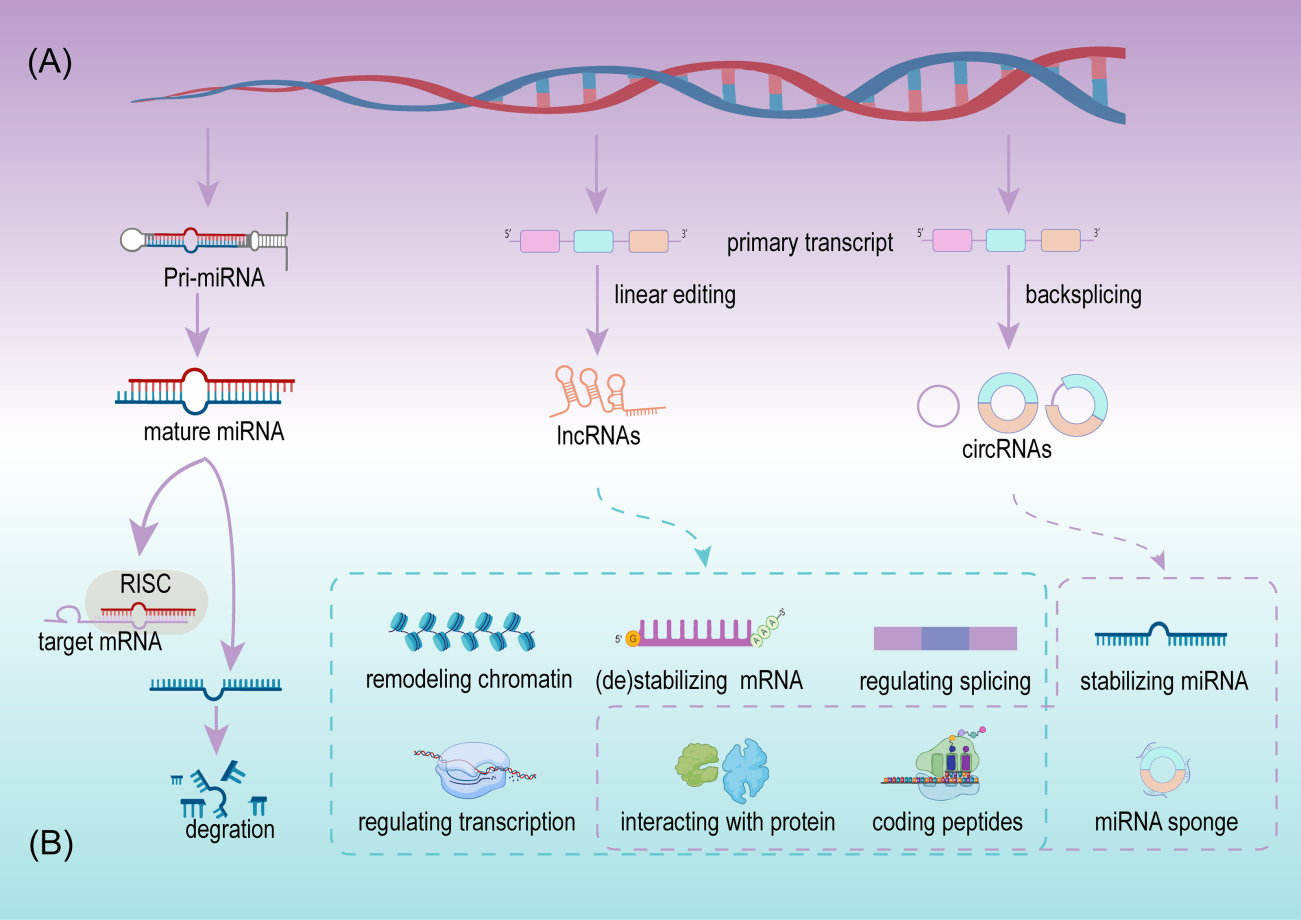
The sketch of biogenesis **(A)** and potential roles **(B)** of miRNA, lncRNA, and circRNA.

MiRNA, deriving from primary miRNAs are ~22 nucleotides in length, and are a dominating type of small RNA (25). Since the first miRNA was detected in *Caenorhabditis elegans*, the discovery of miRNAs has grown considerably. More than 48,000 miRNA sequences from over 200 organisms have been identified and recorded, including over 1,000 miRNAs from humans (26, 27). The biogenesis of miRNA is a complex multi-step process and is regulated at multiple levels (**Figure 2A**). A mature miRNA is initially double stranded. One of the double strands interacts with the RNA-induced silencing complex (RISC), and the remaining strand is eliminated or acts as a miRNA homeostasis regulator after separation from the double strand. Next, RISC silences the target mRNA by matching the binding site (28) (**Figure 2B**). Since the regulation and interaction between miRNA and mRNA is one-to-many, a complex regulatory network is formed (25). miRNAs participate in the regulation of cell growth, development, and pathophysiological processes, including tumors. In tumors, miRNAs serve as promoters or suppressors to a certain extent (29).

With the increase of exosome-related research, exosomal miRNAs have attracted the most attention. According to the available research, four potential pathways are used to compartmentalize miRNAs into exosomes: nSMase2-dependent pathway, miRNA motif and hnRNP-dependent pathway, 3′-end of the miRNA sequence-dependent pathway, and miRISC-related pathway (30). The composition of miRNAs in exosomes not only depends on the properties of the different parent cells but also depends on the different intracellular conditions within the same parent cell (31). Exosomal miRNAs are delivered from the parent cell to the recipient cell and change the biological characteristics of the latter, thereby influencing the destination of tumors. With the exception of the canonical roles of miRNAs, several exosomal miRNAs have been found to play entirely novel roles in recipient cells, such as binding to toll-like receptors (TLRs) and stimulating immune cells (32). Furthermore, exosomal miRNAs are characterized by stability and heterogeneity in urine, blood, and other body fluids, making them promising biomarkers for various types of cancers, including BCa (30).

### Long Noncoding RNA

LncRNAs are defined as a class of transcripts longer than 200 nucleotides with no or limited protein translation potency, but this definition cannot adequately include all lncRNAs (33). Details on the biogenesis and regulation of lncRNAs have not been as clearly as for miRNAs, although, expression of lncRNAs is cell type and disease specific (34). LncRNAs are usually defined by their subcellular location, depending on their function. In the cytoplasm, lncRNAs participate in mRNA stabilization and protein function regulation, while they potentially exert their activity as transcriptional regulators, while in the nucleus they act as mRNA editors (33). Available evidence indicates that lncRNAs are more abundant in the nucleus than in the cytoplasm when compared with miRNAs (35). In cancer initiation and progression, lncRNAs exhibit suppressive and promoting functions *via* multiple regulating levels, including chromatin remodeling, regulating of alternative splicing and transcription at the transcriptional level, stabilizing/destabilizing mRNA, interacting with protein at the post-transcriptional level (36), and coding small peptides (37) (**Figure 2B**). The low expression or overexpression of lncRNAs is associated with many hallmarks of cancer, including cell proliferation, apoptosis, invasion, migration, and tumor microenvironment (TME) formation. New lncRNAs are continuously being discovered to play important roles in BCa.

Interestingly, exosomes also contain numerous specific lncRNAs. Exosomal lncRNAs are closely associated with the initiation and progression of BCa, and accumulate in urine and plasma of patients with BCa, revealing great potential in the diagnoses and treatment of BCa.

### Circular RNA

CircRNAs are a class of noncoding RNA with a closed-loop structure, generated from precursor messenger RNAs (mRNAs) *via* a noncanonical splicing process named backsplicing (38) (**Figure 2A**). CircRNAs were initially considered rare and a result of erroneous splicing by-products with no apparent function in the cell until Thomas et al. proposed and confirmed that circRNAs acted as efficient miRNA sponges (39). Since then, studies on circRNAs have increased significantly, and growing evidence confirms that numerous circRNAs exert important biological functions. Summarizing the current studies, circRNAs play important roles in multiple cell functions, including serving as miRNAs sponges, stabilizing miRNAs, acting as protein sponges or decoys, regulating alternative splicing, and coding proteins (39–43) (**Figure 2B**). Meanwhile circRNAs are cell type, tissue, and disease specific (44). Their aberrant expression is closely related to physiological and pathological processes, especially in cancer (45). Due to their resistance to RNA exonuclease, high stability, and specific expression compared with linear RNAs, circRNAs are more suitable as valuable biomarkers.

Similar to miRNAs and lncRNAs, circRNAs are also highly enriched in exosomes, which was confirmed by Li et al. for the first time using RNA-seq analyses, transmission electron microscopy, and other techniques (46). Li et al. also revealed that the circRNA categories are diverse for different parent cells, indicating the existence of potential sorting mechanisms, and the regulation of the level of related miRNAs in parent cells (46) and the interaction between circRNAs and RNA-related proteins (47) are the potential mechanism. Furthermore, increasing exosomal circRNAs have been identified in urine and serum samples, and their relationships between exosomal circRNAs and cancer, including BCa, have gradually been revealed. Due to the protective double−layered membrane of exosomes, circRNAs along with other bioactive substances achieve further stability and degradation resistance, which are important characteristics of biomarkers (48). However, considerable research is still required on exosomal circRNAs.

## The Biological Roles of Exosome-Derived ncRNAs in BCa

**Figure 3:**
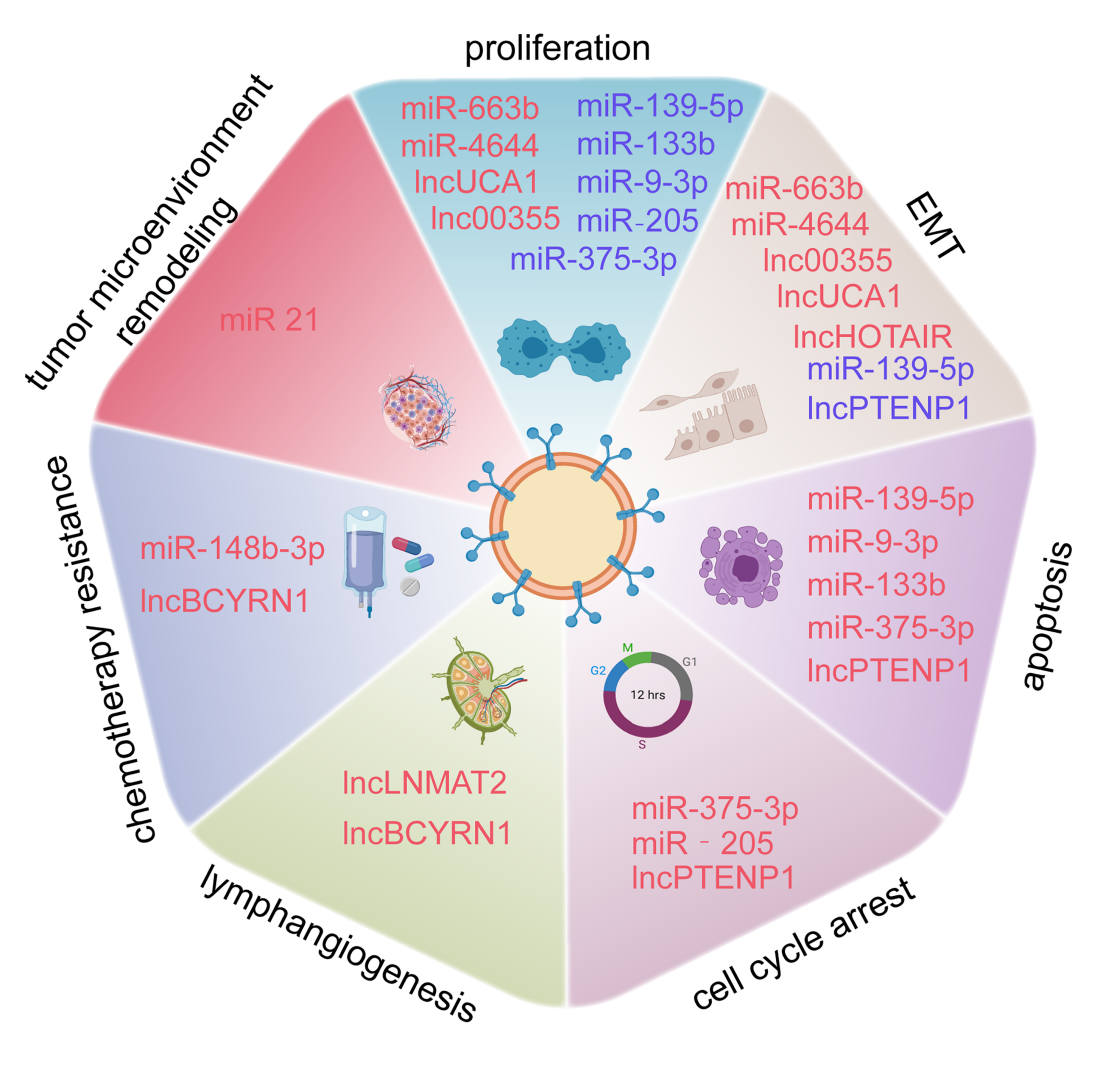
Exosome-derived miRNAs, lncRNAs, and circRNAs are involved in the proliferation, epithelial-mesenchymal transition (EMT), apoptosis, cell cycle arrest, lymphangiogenesis, chemotherapy resistance of BCa, and tumor microenvironment (TME) Remodeling.

BCa, the most frequent malignancy among urinary system tumors, is experiencing a rising incidence worldwide, and has a huge impact on many patients (49). Studies investigating the pathological mechanisms involving BCa are of great help in developing novel treatments; however, research on the molecular mechanisms responsible for BCa still needs to be further strengthened. Exosomes are easily detected in the urine and plasma and are associated with many disease transitions including tumor initiation, progression, and staging, which provide deeper insight into BCa pathogenesis (50). With regard to their roles in exosomes, ncRNAs are a vital class of molecules, which participate in the regulation of tumor proliferation, invasion, migration, apoptosis, cell cycle arrest, angiogenesis, and lymphangiogenesis (**Figure 3**). We summarized the current research to identify the relationship between exosomal ncRNAs and BCa more clearly (**Table 1**).

**Table 1:** The biological functions of exosome-derived ncRNAs in bladder cancer.

ncRNAs	Expression	Parent cell	Target cell	Target gene	Pathway	Biological function	Ref/PMID
miR-21	Up	T24	TAMs	PTEN	PI3K/AKT	Promote cell migration and invasion, mediate immune-suppressive microenvironments	31814034
miR-663b	Up	BCa cells	T24&5637 cells	ERF	–	Promote proliferation and the EMT	31872468
miR-9-3p	Down	BMSCs	UMUC-3	ESM1	MMP-2/9	Inhibit cell proliferation, invasion, metastasis, and promote apoptosis	31734559
miR-139-5p	Down	MSCs	BCa cells	PRC1	–	Inhibit cell proliferation, migration, invasion, and promote apoptosis	33122828
miR-133b	Down	–	–	DUSP1	SAPK/JNK	Inhibit proliferation, promote apoptosis	32627968
miR-4644	Up	–	BCa cells	UBIAD1	–	Promote proliferation, migration, and invasion	33194029
miR-375-3p	Down	T24 cell	T24 cell	FZD8	Wnt/β-catenin	Inhibit proliferation and migration, promote apoptosis, arrest the cell cycle	32716585
miR‐205	–	hWJMSCs	RT4 and T24 cells	–	–	Inhibit proliferation, cell viability, and arrest cell cycle	30387205
miR-148b-3p	Down	CAFs	BCa cells	PTEN	Wnt/β-catenin	Promote the metastasis, EMT, and drug resistance	33423167
lncRNA BCYRN1	Up	BCa cells	HLECs	WNT5A	VEGF-C/VEGFR3	Promote tube formation and migration of HLECs *in vitro*, accelerate lymphangiogenesis, and LN metastasis *in vivo*.	
lncRNAUCA1	Up	5637 cells	UMUC2 cells	–	–	Promote cell proliferation, migration and invasion, tumor growth and progression	28841829
lncRNA PTENP1	Down	HEK 293A	EJ and J82	PTEN	miR-17/PTEN	Promote cell apoptosis, inhibit cell invasion and migration	30285771
lncRNA LNMAT2	Up	BCa cells	HLECs	PROX1	hnRNPA2B1/H3K4	Stimulated HLEC tube formation and migration *in vitro* and enhanced tumor lymphangiogenesis and LN metastasis *in vivo*	31593555
LINC00355	Up	CAFs	BCa cells	–	–	Promote cell proliferation, migration, and invasion	31749189
LINC00355	Up	CAFs	BCa cells	ABCB1	miR-34b-5p/ABCB1	Promote cell resistance to cisplatin	33720323
LINC00960 and LINC02470	Up	High-grade BCa cells/T24 and J82	Low-grade BCa cells/TSGH-8301	–	β-Catenin/TCF4, Notch/HES1, Smad2/3	Promote the EMT and aggressiveness of BCa Cells	32517366
lncRNA HOTAIR	Up	TCC-SUP and T24	TCC-SUP and T24	SNAI1, etc.	HOTAIR/EMT	Promote migration and invasion	26800519
CircPRMT5	Up	UCB cells	UCB cells	miR-30c	circPRMT5/miR-30c/SNAIL1/E-cadherin	Promote metastasis, EMT, and aggressiveness	30305293

TAMs, tumor-associated macrophages; PTEN, phosphatase and tensin homolog; ERF, Ets2-repressor factor; BMSCs, bone marrow-derived mesenchymal stem cells; MSCs, mesenchymal stem cells; hWJMSCs, human umbilical cord Wharton jelly mesenchymal stem cell; CAFs, cancer-associated fibroblasts; HLECs, human lymphatic endothelial cells.

### Exosome-Derived ncRNAs and Proliferation

Uncontrolled cell proliferation is one of the main characteristics of all tumors. Comparing normal and tumor tissue samples in microarray analysis, the aberrant expression of genes associated with cell proliferation is the most common case (51). Identifying the molecular mechanisms involved in tumor cell proliferation can bring new hope for tumor diagnosis and treatment. Exosomal ncRNAs have been confirmed to be involved in regulating the proliferation of BCa cells.

Numerous miRNAs play a crucial role in promoting or inhibiting cell proliferation. Exosomal miR-663b circulates in the plasma of BCa patients. Overexpression of miR-663b promotes cell proliferation and tumor development at an significant level by targeting Ets2-repressor factor (ERF), while upregulating the ERF can eliminate the promotion function of miR-663b (52). Exosomal miR-4644 is another miRNA that promotes cell proliferation. Yan et al. pointed out that compared with non-BCa patients, the level of miR-4644 is higher in plasma exosomes of BCa patients. It directly targets and downregulates the expression of UbiA prenyltransferase domain-containing protein 1 (UBIAD1), and then stimulating BCa cell proliferation (53). Conversely, miR-139-5p and miR-133b are downregulated in serum exosomes and inhibited the BCa cell multiplication (54, 55).

Exosomal lncRNAs, such as lnc_00355 and lncRNA-UCA1, are involved in the process of controlling the proliferation of BCa cells. Lnc_00355, produced by cancer-associated fibroblasts, can be detected in the urine. It was internalized by BCa cells and functioned as a tumor suppressor (56). Another study by Xue et al. revealed that lncRNA-UCA1 in serum exosomes are generated by BCa 5637 cells and taken in by BCa UMUC2 cells, then impeding the proliferation of the latter (57).

Since the research on exosomal circRNAs in BCa is still in the initial phases, no circRNAs have been found that regulate cell proliferation. Based on the above evidence, ncRNAs, especially miRNAs and lncRNAs, are involved in crucial regulation of cell proliferation of BCa.

### Roles of Exosome-Derived ncRNAs in the Regulation of EMT Signaling Networks in the Invasion-Metastasis Cascade

**Figure 4:**
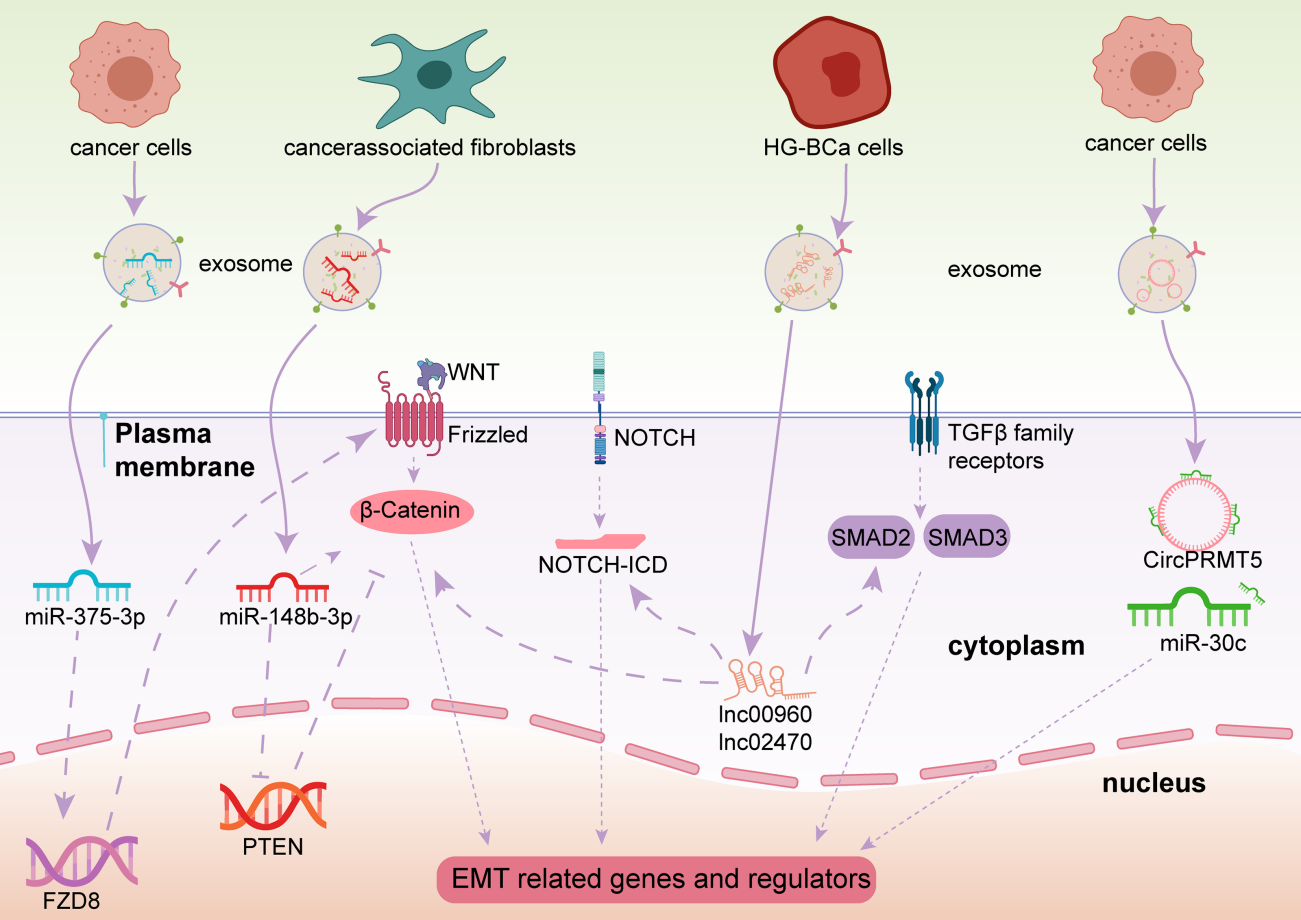
Exosome-derived ncRNAs play important roles in regulating the EMT process *via* different pathways in BCa.

EMT is a well-known cellular program associated with malignant progression. Cancer cells achieve greater tumor initiation, invasion, and metastasis capacities and stronger resistance to several therapeutic regimens through EMT (58). EMT consists of a series of multiple and dynamic shifting states rather than a simple and binary condition, whose processes can be reversed in certain conditions through a mechanism called mesenchymal to epithelial transition (MET) (59). Epigenetics is a vital theory used to explain the regulation of EMT (60). In contrast to the direct effects induced by EMT transcription factors on gene expression, ncRNAs, as an epigenetic regulator of EMT, and regulate EMT progression indirectly (60, 61), including *via* the PI3K/Akt signaling pathway, Wnt/β-catenin signaling pathway, and NOTCH pathway. Exosomal ncRNAs have also been implicated in EMT (**Figure 4**).

In the Wnt/β-catenin pathway, and specifically in canonical Wnt signaling, once the binding of Wnt and Frizzled receptor is complete, β-catenin is prevented from degradation, then β-catenin enters the nucleus and activates EMT-related transcription factors (EMT-TFs) (62). As a result, E-cadherin, a vital element for epithelial cell-to-cell adhesion, is absent and the EMT process is launched (63). Some exosomal miRNAs have been identified to initiate or prevent EMT processes in BCa through the Wnt/β-catenin signaling pathway. Exosomal miR-148b-3p is secreted by cancer-associated fibroblasts and is transmitted into BCa cells and acts as a tumor promotor to trigger EMT initiation by interacting with the PTEN gene (64). Unlike miR-148b-3p, Li et al. revealed that exosomal miR-375-3p is a tumor suppressor. By directly restraining Frizzled-8 (FZD8) gene expression, miR-375-3p blocks the Wnt/β-catenin pathway as well as its downstream molecules to inhibit the EMT process (65).

Other pathways besides the EMT signaling pathways mentioned above, Notch signaling and Smad2/3 signaling are also associated with EMT processes in BCa. Huang et al. pointed out that exosomal lnc_00960 and lnc_02470 promoted EMT and the aggressiveness of BCa cells. They treated BCa TSGH-8301 cells with exosomes derived from BCa T24 or J82 cells, leading to decreased E-cadherin levels and increased N-cadherin, vimentin, MMP2, and MMP9 levels in TSGH-8301 cells (66). Berrondo et al. also reported that exosomal lncHOTAIR was associated with low expression of SNAI1, LAMB3, and LAMBC2 mRNA, which affect the EMT process of BCa cells (67). It was confirmed that miR-30c directly bound to the 3′-UTR of SNAI1 to suppress the EMT procedure of esophageal squamous cell carcinoma (68), Chen et al. uncovered that circPRMT5 was involved in circPRMT5/miR-30c/SNAIL1/E-cadherin pathway in an intriguing way. CircPRMT5 entered into the urothelial carcinoma of the bladder (UCB) cells through exosomes and blocked the physiological effects of miR-30c by acting as a circRNA sponge. Then the SNAIL1 and its downstream elements E-cadherin were reduced. More interestingly, knockdown or overexpression of circPRMT5 does not affect its host gene, indicating circPRTM5 functioned in a PRMT5-independent way (69).

Moreover, Lin et al. found that exosomal miR-21 is enriched in the urine of BCa patients. When T24 cell-derived exosomes containing miR-21 are engulfed by tumor-associated macrophages, PTEN suppression, and PI3K/AKT−STAT3 pathway activation were observed *in vitro*, which promoted M2 macrophage polarization and BCa tumorigenesis (70). Exosomal miR-21 is a popular molecule. Extensive studies have found that it is associated with a variety of cancers, such as ovarian cancer, breast cancer, and prostate cancer (71). Among these, miR-21 was confirmed to be involved in regulating EMT in breast cancer A549 cells (72). Although the PI3K/AKT pathway is an important EMT activating signal related to bladder carcinogenesis, whether miR-21 participates in EMT regulation in BCa still needs verification.

### Exosome-Derived ncRNAs and Apoptosis

Apoptosis is an important mechanism involved in cellular development, maintenance, and elimination (73). It is a programmed and controlled process of active cell destruction, which requires complex cellular signal regulation and multiple changes in cell morphology as well as molecular changes (74, 75). Two major apoptotic pathways are widely accepted. In the death-receptor pathway, the death-receptor superfamily triggers the former, leading to the formation of a death-inducing signaling complex and the activation of caspase-8 (76). In the mitochondrial pathway, cytochrome *c* is a core component. Cytochrome *c* interacts with Apaf-1 and combines with procaspase-9 to form the apoptosome which induces cell apoptosis (76).

In recent studies, exosomal ncRNAs have been increasingly been reported to participate in the regulation of BCa cell apoptosis. Dual-specificity protein phosphatase 1 (DUSP1) has been shown to inhibit cancer progression through the SAPK/JNK signaling pathway (77). Cai et al. found exosomal miR-133b could promote the apoptosis of 5637 and T24 cells in a cell apoptosis assay by targeting DUSP1, and by significantly reducing tumor burden in the xenograft mouse model (55). Exosomal miR-9-3p produced by bone marrow-derived mesenchymal stem cells (BMSCs) participate in the regulation of BCa UMUC-3 cell apoptosis and by targeting endothelial cell-specific molecule 1 (ESM1) (78). It is worth noting that ESM1 was known for its angiogenic and inflammatory properties (79) and was found at high expression levels in blood vessels in invasive bladder cancer tissues (80). However, whether exosomal miR-9-3p mediates the angiogenesis in BCa is inconclusive (78). Another study conducted by Xu et al. revealed that miR-29c induced the apoptosis of bladder cancer BIU-87 cells by downregulating two well-known antiapoptotic genes, BCL-2 and MCL-1 (81). Exosomal miR-139-5p and miR-375-3p also expedited cell apoptosis by targeting PRC1 and FZD8, respectively (54, 65). In addition to miRNAs, exosomal lncRNA PTENP1 accelerates the bladder cancer EJ and J82 cell apoptosis (82). However, the exploration of exosomal circRNAs in the apoptosis of BCa cells is still blank.

It is worthy of note that most results mentioned above have been verified by cell apoptosis assays, although the underlying mechanisms remain to be clarified.

### Exosome-Derived ncRNAs and Cell Cycle Arrest

Cell cycle arrest is tightly related to different pathophysiological mechanisms, including aging, atherosclerosis, and osteoarthritis (83). Dysregulation of the cell-cycle is an elemental feature of tumorigenesis and tumor progression. The most prominent mechanism is cell cycle arrest induced by p53 (84, 85). Reversible cell cycle arrest is the mechanism through which cancer cells enter a dormant transition state, an important condition used by tumor cells to acquire additional mutations, ensure survival, and metastasize, as well as to develop treatment-resistance (86).

Recent studies have reported that exosomal ncRNAs participate in BCa cell cycle arrest and in the control of progression of BCa (65, 87). A miR-375-3p mimic can arrest the cell cycle of T24 cells at G1 *in vitro*, and thereby reduce cell proliferation and clone formation significantly (65). Zhang et al. extracted exosomes containing miR-205 from human umbilical cord Wharton jelly mesenchymal stem cell (hWJMSCs) and transferred them into human bladder transitional cell papilloma RT4 cells and T24 cells. They found that the number of RT4 and T24 cells at S-phase distinctly increased and cyclin J (CCNJ) was the target gene of miR-205 (87).

Besides exosomal miRNAs, exosomal lncRNAs are also involved in cell cycle arrest. Exosomal lncRNA-PTENP1 functions as a tumor suppressor by promoting cell cycle arrest of J82 cells in the G2 phase and of EJ cells in the S and G2 phases, respectively (82). Nonetheless, the associations between exosomal circRNAs and BCa cell cycle regulation are still unclear. Greater attention should be paid to exosomal circRNAs to better understand their role in BCa pathogenesis.

### Exosome-Derived ncRNAs and Lymphangiogenesis

Many studies have investigated the role of angiogenesis in tumor formation, but the mechanisms underlying lymphangiogenesis are still insufficient, despite the great progress in recent years (88). Lymphangiogenesis may occur in several pathophysiological processes including wound healing, inflammation, and tumor metastasis. The main lymphangiogenesis-specific markers studied to date include vascular endothelial growth factor (VEGF), LYVE-1, and PROX-1 (88). VEGF is one of the most canonical and important markers, some studies have indicated that miR-128 and lncBLACAT2 have directly targeted the VEGF-C to mediate lymphangiogenesis in BCa *in vivo* and *in vitro* (89, 90). In tumors, lymph node metastasis is an important process of tumor progression, as well as an important reference for tumor staging and treatment options. Tumor cells can accelerate progression into the lymphatic vessels by stimulating the germination and expansion of peritumoral lymphatic vessels by growth factors (91, 92). Overall, cancer-associated lymphangiogenesis is no doubt a promising prognostic candidate for predicting the risk of lymph node metastasis in various cancers (93, 94).

Park et al. reported clearly that exosomes could serve as communication vehicles between the lymphatic system and BCa, and tumor-derived exosomes may potentially remodel the lymphatic system horizontally by transferring epigenetic and genetic information (95). Chen et al. uncovered a significant finding in 2019 when investigating tumor lymphangiogenesis regulated by exosomal ncRNAs. They found that BCa cell-derived exosomal lncLNMAT2 can mediate lymphangiogenesis and promote lymph nodes metastasis in BCa. Different from the canonical VEGF-C dependent mechanism, this function did not rely on VEGF-C. The author’s confirmed these findings using both *in vivo* and *in vitro* models (96). Exosomal lncBCYRN1 was also confirmed by Zheng et al. (97) to participate in the lymphangiogenesis of BCa. Their research showed that exosomal lncBCYRN1 and lncLNMAT2 have similar functions *in vivo* and *in vitro*, and both are associated with H3K4 trimethylation (97). In addition, no exosomal miRNA or circRNA has been reported to interact with regulation networks of lymphangiogenesis at the time of preparation of this review.

### Exosome-Derived ncRNAs and Chemotherapy Resistance

The treatment and therapy resistance of malignant tumors have always plagued clinicians. Many measures have been used for the treatment of BCa. In addition to surgical treatment, chemotherapy is a crucial procedure able to reduce tumor recurrence in patients with BCa. Although TaT1 BCa can be eradicated by TURBT, intravesical chemotherapy is still a routine treatment capable of preventing tumor recurrence or progression to MIBC (3). Immediate single instillation had been confirmed to reduce the 5-year recurrence rate up to 14% (98). Except for immediate single instillation, some TaT1 BCa patients still require extra adjuvant intravesical therapy according to prognosis (3). Muscle-invasive and metastatic BCa patients, can obtain potential benefit from cisplatin-based pre-operative neoadjuvant chemotherapy (99, 100), and postoperative chemotherapy is also recommended for specific patients. Chemotherapy has achieved a prominent position in the treatment of BCa.

However, some BCa patients are not inherently chemosensitive or may acquire resistance after intensive treatment and may ultimately lead to treatment failure. Research on the mechanisms underlying chemotherapy resistance can improve the effectiveness of existing treatment options and can provide clinicians with potential new treatment options. By examining the sensitivity of BCa cells to Paclitaxel (PTX) and doxorubicin (DOX), Shan et al. demonstrated that after treatment, exosomes derived from cancer-associated fibroblasts, T24 and 5367 cells, showed lower levels of apoptosis, indicating exosomal miR-148b-3p contributed to the reduction in chemosensitivity (64). Liang et al. further studied exosomal lnc00355 produced by cancer-associated fibroblasts (CAFs) and found that they bind miR-34b-5p through a ceRNA mechanism to up-regulate the expression of ABCB1, thereby participating in the cisplatin resistance of BCa (101). Further research is required to better understand the mechanism by which exosomal ncRNAs regulate the chemosensitivity of BCa.

### Exosome-Derived ncRNAs and TME Remodeling

The TME is a complex and dynamic system that contains cellular and noncellular components. Tumor survival and progression depend greatly on the TME, and tumor cells in turn can remodel the TME to make it more suitable for tumor cell survival. The convoluted communication network, including autocrine and paracrine signaling, is the cornerstone of regulating the TME. Among them, exosomal ncRNAs packaged by tumor cells can participate in the process of immune cell maturation and differentiation, remodeling the optimum TME for tumor cells, and creating an immunosuppressive microenvironment.

Lin et al. confirmed that exosomal miR−21 packaged by BCa cells could promote M2 phenotypic polarization *via* the PI3K/AKT pathway in macrophages. By polarizing tumor−associated macrophages, migration, and invasion of T24 cells were enhanced. In other tumors, exosomal ncRNAs have also been confirmed to be involved in the regulation of the TME. Exosomal miR-10a and miR-21 influence the expansion and function of myeloid-derived suppressor cells in glioma (102). In liposarcoma, exosomal miR-25-3p and miR-92a-3p produced by liposarcoma cells could induce tumor-associated macrophages to secrete IL6 *via* TLR7/8-dependent pathway, thereby enhancing the proliferation and invasion of liposarcoma cells (103). Breast cancer cells deliver lncRNA SNHG16 through exosomes to induce γδ1 Treg cells to upregulate the expression of CD74 (104). The metastasis of nonsmall cell lung cancer can be promoted when nonsmall cell lung cancer cells transfer exosomal circFARSA to mediate M2 macrophage polarization through the PTEN/PI3K/AKT axis (105). Overall, exosomal ncRNAs participate in the suppression of the immune microenvironment in a variety of tumors, thereby promoting tumor progression. However, in BCa, further evidence is required.

## Clinical Significance of Exosome-Derived ncRNAs in BCa

**Figure 5:**
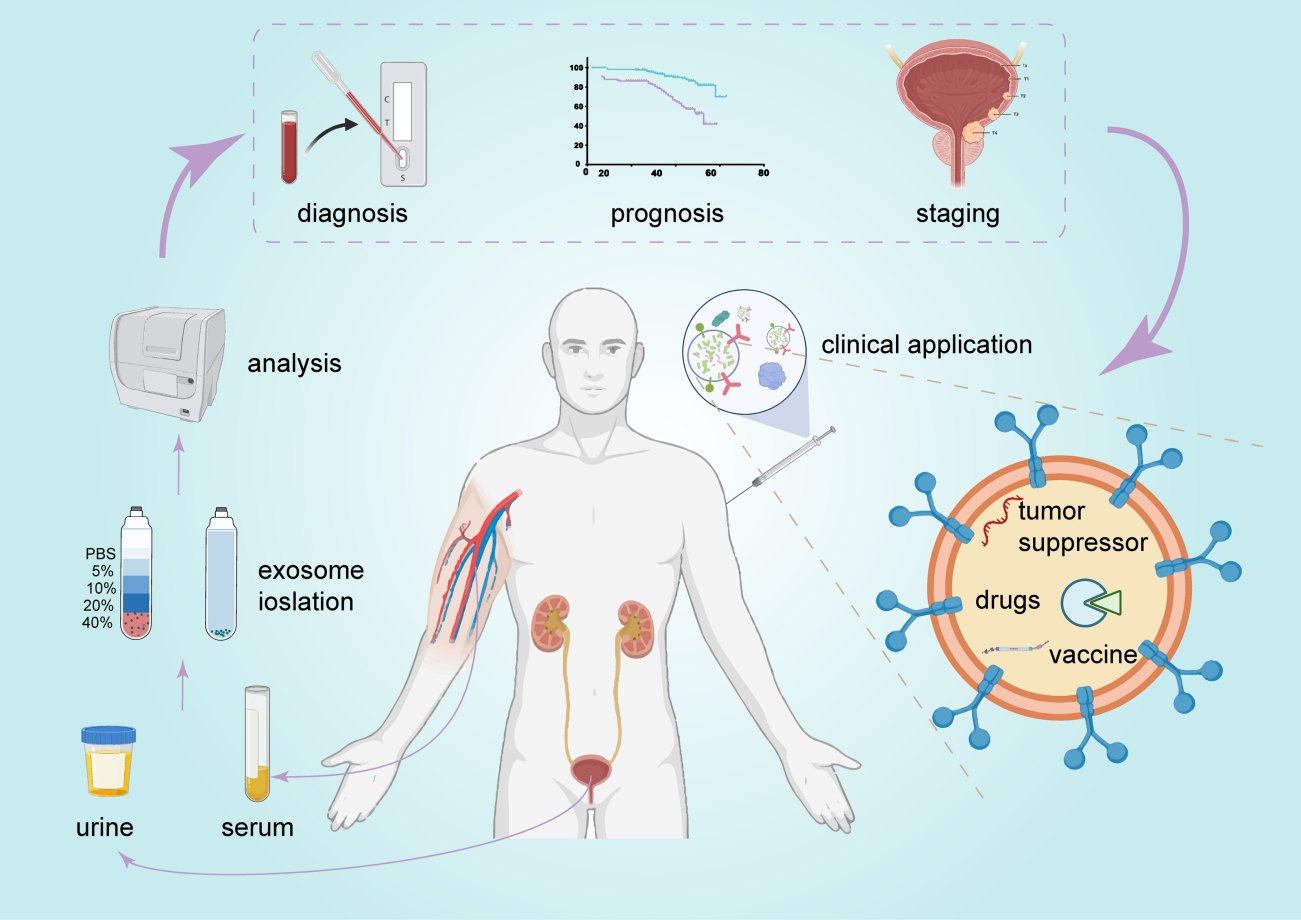
The promising clinical application of exosomes in BCa. Exosomes can be detected in urine and serum and serve as promising biomarkers for BCa diagnosis and prognosis. They are also a potential candidate for BCa therapy.

BCa is characterized by high morbidity and mortality and requires improvements in diagnosis, treatment, and prognosis stratification. A variety of functional molecules has been identified in exosomes and reflects the complex heterogeneity of the entire tumor. Moreover, exosomes are very stable and can be found in almost all types of human biological fluids (11), including urine and serum. These factors make exosomes very promising biomarkers. As mentioned above, exosomal ncRNAs are involved in various signaling pathways and play important regulatory roles in BCa. Therefore, targeting exosomes or their cargo may be a promising therapeutic option in the treatment of BCa (**Figure 5**).

### Relationships Between Exosome-Derived ncRNAs and Clinicopathologic Characteristics in BCa

Exosomal ncRNAs have been reported to both correlate and not correlate with several clinicopathological parameters in BCa (**Table 2**). CircPRMT5 was significantly upregulated in serum exosomes in patients with BCa. In addition to lymph node metastasis (LNM), circPRMT5 was positively correlated to advanced tumor progression but not with sex or age (69). Abbastabar et al. found that urine exosomal lncRNA ANRIL and lncRNA PCAT-1 were not related with tumor size, tumor grade, tumor stage, or recurrence (106). However, the relationship between exosomal lncRNA PCAT1 and tumor grade, stage, and recurrence of BCa has been controversial across different studies. Zhan et al. found that urine exosomal lncRNA PCAT1 was closely associated with tumor stage and recurrence in patients with BCa, but not with tumor grade, LNM, age, sex, and other clinical characteristics (107). In another study, serum exosomal lncRNA PCAT1 was associated with tumor grade (108). Nonetheless, in these studies, differences in relationships between exosomal lncRNA PCAT1 and clinical parameters of BCa may be caused by the heterogeneity of samples and of research methods. In addition, Chen et al. found that the expression of urinary exosomal lncRNA LNMAT2 was upregulated, which was positively correlated with LNM and distant metastasis but not with tumor grade, stage, age, and sex (96). In fact, exosomal lncRNA LNMAT2 is the only exosomal ncRNA associated with distant metastasis in the currently available studies. The expression of serum exosomal lncRNA H19 of BCa patients was significantly higher than that in normal volunteers, and its expression was positively correlated with LNM (109). Moreover, other exosomal ncRNAs, such as lncRNA MALAT1 (107), lncRNA SPRY4-IT1 (107), lncRNA UBC1 (108), and lncRNA SNHG1 (108), can also be detected in the urine or blood of BCa patients, and are associated with various clinicopathologic characteristics of BCa.

**Table 2:** Relationship between exosome-derived ncRNAs and clinicopathologic characteristics in BCa.

Type of ncRNAs	Exosomal ncRNAs	Clinical sample	Dysregulation	Number of patients	Gender	Age	Tumor size	Tumor grade	T stage	LMN	DM	Number of tumors	Recurrence	Ref./PMID
CircRNA	circPRMT5	Serum	Up	71	No	No	–	–	Yes	Yes	–	–	–	30305293
LncRNA	ANRIL	Urine	Up	30	–	–	No	No	No	–	–	–	No	32231490
LncRNA	BCYRN1	Urine	Up	210	No	No	–	No	Yes	Yes	–	–	–	34323412
LncRNA	PCAT-1	Urine	Up	30	–	–	No	No	No	–	–	–	No	32231490
LncRNA	MALAT1	Urine	Up	80	No	No	–	No	No	No	–	–	Yes	30268126
LncRNA	PCAT-1	Urine	Up	80	No	No	–	No	Yes	No	–	–	Yes	30268126
LncRNA	SPRY4-IT1	Urine	Up	80	No	No	–	No	Yes	No	–	–	–	30268126
LncRNA	H19	Serum	Up	52	No	No	–	No	Yes	Yes	–	–	–	30576305
LncRNA	LNMAT2	Urine	Up	266	No	No	–	No	No	Yes	Yes	–	–	31593555
LncRNA	PCAT-1	Serum	Up	160	No	No	–	Yes	No	No	–	–	–	30467945
LncRNA	UBC1	Serum	Up	160	No	No	–	No	Yes	Yes	–	–	–	30467945
LncRNA	SNHG16	Serum	Up	160	No	No	–	No	Yes	No	–	–	–	30467945

BCa, bladder cancer; ncRNAs, noncoding RNAs; circRNA, circular RNA; LncRNA, long noncoding RNA; LNM, lymph nodes metastasis; NAT, adjacent noncancerous tissues; LNM, lymph node metastasis; DM, distant metastasis.

To summarize the current studies, common clinical parameters related to exosomal ncRNAs include tumor grade, tumor stage, LNM, and distant metastasis. Existing evidence shows that age, sex, and tumor size show no significant relationship with exosomal ncRNAs. It is worth noting that the number of lesions is also an important parameter of BCa, but no studies to date have evaluated the relationship between the number of BCa lesions and exosomal ncRNAs.

### Exosome-Derived ncRNAs Serve as Diagnostic Biomarkers for BCa

Cystoscopy and biopsy are currently essential methods for the diagnosis of BCa, but they are uncomfortable, costly, and invasive (49, 110). In addition, nuclear matrix protein 22 (NMP22) and bladder tumor antigen (BTA) currently used as diagnostic biomarkers are not suitable for low-grade BCa due to the lack of diagnostic specificity and sensitivity (111). Therefore, it is still of great importance to define novel molecular targets for the early diagnosis of BCa.

Exosomal ncRNAs can be detected in the serum and urine of patients, indicating their potential as diagnostic biomarkers (**Table 3**). For example, the expression of serum exosomal lncRNA UCA1 in BCa patients was higher than that in the control group, and the area under the curve (AUC) value was 0.878, indicating their good diagnostic efficiency for BCa diagnosis (57). Similarly, Yazarlou et al. reported that urine exosomal lncRNA UCA1-201 and lncRNA UCA1-203 were very effective in distinguishing BCa patients from healthy individuals, as their AUC were 0.93 and 0.8, respectively (112). The diagnostic value of exosomal lncRNA PCAT-1 in BCa has attracted much attention. Zhang et al. reported that the AUC of serum exosomal lncRNA PCAT-1 was 0.753 (108). In two other studies, the AUCs of urine exosomal lncRNA PCAT-1 were 0.832 and 0.729, respectively (106, 107). Overall, these results indicate a clear diagnostic value of exosomal lncRNA PCAT-1 in BCa.

**Table 3:** Exosome-derived ncRNAs serve as potential diagnostic biomarkers for BCa.

Type of ncRNAs	Exosomal ncRNAs	Types of specimen	Sample size		Dysregulation	AUC	95% CI	Sensitivity	Specificity	Ref./PMID
			Case	Control						
LncRNA	UCA1-201	Urine	59	24	Down	0.93	–	75.4%	100%	30568497
LncRNA	UCA1-203	Urine	59	24	Up	0.8	–	72.4%	75%	30568497
LncRNA	MALAT1	Urine	59	24	Up	0.73	–	63.8%	83.3%	30568497
LncRNA	Lnc_00355	Urine	59	24	Up	0.75	–	68%	79.2%	30568497
LncRNA	Combination of UCA1-201, UCA1-203, MALAT1, and LINC00355	Urine	59	24	–	0.96	–	92%	91.7%	30568497
LncRNA	ANRIL	Urine	30	10	Up	0.723	0.498–0.948	46.7%	87.5%	32231490
LncRNA	PCAT-1	Urine	30	10	Up	0.729	0.495–0.963	43.3%	87.5%	32231490
LncRNA	UCA1	Serum	30	30	Up	0.878	0.7926–0.964	80%	83.3%	28841829
LncRNA	PTENP1	Plasma	50	60	Down	0.743	0.645–0.840	–	–	30285771
LncRNA	Combination of MALAT1, PCAT-1, and SPRY4-IT1	Urine	104	104	Up	0.854	0.799–0.899	70.2%	85.6%	30268126
LncRNA	MALAT1	Urine	104	104	Up	0.844	0.787–0.890	72.1%	84.6%	30268126
LncRNA	PCAT-1	Urine	104	104	Up	0.832	0.774–0.880	72.1%	81.7%	30268126
LncRNA	SPRY4-IT1	Urine	104	104	Up	0.760	0.696–0.817	66.3%	76.9%	30268126
LncRNA	H19	Serum	52	52	Up	0.851	0.787–0.903	74.07%	78.08%	30576305
LncRNA	LNMAT2	Urine	206	120	Up	0.769	0.715–0.823	–	–	31593555
LncRNA	Combination of PCAT-1, UBC1, and SNHG16	Serum	160	160	Up	0.826	0.780–0.866	80%	75%	30467945
LncRNA	PCAT-1	Serum	160	160	Up	0.753	–	–	–	30467945
LncRNA	UBC1	Serum	160	160	Up	0.751	–	–	–	30467945
LncRNA	SNHG16	Serum	160	160	Up	0.681	–	–	–	30467945
microRNA	Combination of miR-139-5p, miR-136-3p and miR-19b1-5p	Urine	59	34	–	0.903	–	80%	88.2%	31679136

BCa, bladder cancer; AUC, area under the receiver-operating characteristic curve; CI, confidence interval.

Although single exosomal ncRNA can diagnose BCa, a combined biomarkers set may predict BCa more accurately than a single biomarker. Some studies have explored the combined application of exosomal ncRNAs in the diagnosis of BCa. For example, the combination of blood exosomal lncRNA PCAT-1, UBC1, and SNHG16 had an AUC of 0.826, a sensitivity of 80%, and a specificity of 75% (108). The combined application of urine exosomal miR-139-5p, miR-136-3p, and miR-19b1-5p increased the AUC to 0.903, sensitivity to 80%, and specificity to 88.2% (113). The combination of lncRNA UCA1-201, UCA1-203, MALAT1, and Lnc_00355 effectively improved the diagnostic performance with an AUC of 0.96, a sensitivity of 92%, and a specificity of 91.7% (112). This is the most appropriate set of biomarkers of exosomal ncRNAs identified for the diagnosis of BCa.

### Exosome-Derived ncRNAs Serve as Prognostic Biomarkers for BCa

Exosomal ncRNAs can also be used to predict patient survival parameters, such as cancer-specific mortality (CSM), recurrence-free survival (RFS), overall survival (OS), and disease-free survival (DFS). To further analyze the prognostic value of exosomal ncRNAs in BCa, we collected information from studies reporting survival information to evaluate the relationship between exosomal ncRNAs expression and RFS, OS, and DFS. To date, only nine exosomal ncRNAs have been identified as having prognostic value as biomarkers (**Table 4**).

**Table 4:** Exosome-derived ncRNAs serve as potential prognostic biomarkers for BCa.

Type of ncRNAs	Exosomal ncRNAs	Types of specimen	Number of patients		Survival outcomes	*p*-Value	Hazard ratio	95% CI	Ref./PMID
			High	Low					
LncRNA	BCYRN1	Urine	105	105	DFS	0.015	1.563	1.091–2.239	34323412
LncRNA	LNMAT2	Urine	103	103	DFS	<0.05	0.57	0.38–0.81	31593555
LncRNA	BCYRN1	Urine	105	105	OS	0.004	1.781	1.199–2.646	34323412
LncRNA	H19	Serum	26	26	OS	0.006	2.193	1.284–3.698	30576305
LncRNA	LNMAT2	Urine	103	103	OS	<0.05	0.5	0.33–0.74	31593555
LncRNA	PCAT-1	Urine	40	40	RFS	0.001	6.368	1.375–29.496	30268126
LncRNA	MALAT1	Urine	40	40	RFS	0.002	3.627	1.483–8.872	30268126
LncRNA	UBC1	Serum	37	37	RFS	0.01	2.371	1.157–4.857	30467945
MicroRNA	miR-185-5p	Plasma	40	5	CSM	0.001	–	–	32527011
MicroRNA	miR-106a-5p	Plasma	40	5	CSM	0.039	–	–	32527011
MicroRNA	miR-10b-5p	Plasma	40	5	CSM	0.047	–	–	32527011

BCa, bladder cancer; ncRNAs, noncoding RNAs; RFS, recurrence-free survival; OS, overall survival; DFS, disease-free survival; CSM, cancer-specific mortality survival; CI, confidence interval.

Kaplan-Meier survival analysis showed that higher expression of urine exosomal lncRNA PCAT-1 and MALAT1, as well as serum exosomal lncRNA UBC1 was associated with worse RFS in patients with BCa (107, 108), while patients with higher serum exosomal lncRNA H19 expression had lower OS (109). Additionally, low expression of serum exosomal miR-185-5p and miR-106a-5p or high expression of miR-10b-5p showed shorter survival (114). Notably, Chen et al. reported that urine exosomal lncRNA LNMAT2 was closely related to OS and DFS, with hazard ratio (HR) values of 0.5 and 0.57, respectively, which seemed to indicate that LNMAT2 was a protective factor (96). Nonetheless, the authors also showed that exosomal LNMAT2 promoted LNM of BCa. Thus, the relationship between exosomal LNMAT2 and BCa remains to be clarified, and more evidence or a larger sample size may be needed for further confirmation.

### Therapeutic Potential of Exosome-Derived ncRNAs in BCa

With the further understanding of the functions of ncRNAs, exosomal ncRNAs have gradually become an attractive therapeutic target for the treatment of BCa. The role of exosomal ncRNAs in the development and progression of BCa is two-sided. Tumor-derived exosomes can carry oncogenic ncRNAs into recipient cells to promote tumor progression, while some normal cell-derived exosomes can carry tumor suppressor ncRNAs to inhibit tumor progression. Thus, researchers can achieve therapeutic goals by controlling the biogenesis, delivery, and uptake of exosomes. Nishida-Aoki et al. reported that antibody treatment targeting cancer-derived exosomes could reduce breast cancer metastasis (115). To date, no similar study has been reported in BCa. Exosomes can be used as delivery vehicles to target cancer cells due to their small size and good histocompatibility. They can cross biological barriers, such as vascular endothelial barriers. Therefore, we can package tumor-suppressive ncRNAs into exosomes through electroporation or transfection, and then transfer the exosomes into BCa cells to reverse the malignant phenotype of the latter. A study found that BCa cells were more likely (≥50 times) to absorb exosomes than normal urethral epithelial cells, and the delivery of Small interfering RNA (siRNA) *via* exosomes successfully suppressed PLK-1 expression in BCa cells (116). Exosomes can also contain pharmacological drugs (117). Several studies have shown that therapeutic nanoparticles can be loaded into liposomes, which can increase drug concentrations in target tissues and reduce toxic effects in normal tissues (118).

Exosomes can also be used as cancer vaccines. Tumor-derived exosomes meet all antigen presentation criteria as cell-free vaccines (119). ncRNA are a key regulator of the immune system, affecting the differentiation, maturation, expansion, and function of immune cells (120). Therefore, ncRNAs-modified exosomes can be used for immunotherapy of BCa. For example, tumor-derived exosomes modified with specific ncRNAs can target IL-6, IL-17, IL-1B, TGFβ, IFN-γ, and TLR4 to induce dendritic cell maturation and to enhance its immune-stimulating capacity (121).

Overall, exosomal ncRNAs are expected to play important roles in the treatment of BCa. However, research in this area is still very limited, and more efforts are needed to explore exosomal ncRNAs-based therapies for patients with BCa.

## Conclusions and Outlook

BCa is a common urinary system tumor with a poor prognosis and weak therapeutic reactivity. The therapeutic schedule for BCa has not changed significantly in time, and the current survival rates still rely on an insufficient understanding of the underlying molecular mechanisms of BCa. ncRNAs, especially miRNAs and lncRNAs, have been found to be ubiquitously and differentially expressed in BCa tissues, urine, and blood. Their dysfunction is tightly related to BCa initiation and progression. Exosomes are a class of extracellular vesicles containing abundant molecular components, including ncRNAs. Exosomes complete cell-to-cell communication and regulate the biological functions of the recipient cells by transporting the components derived from the parent cells. An increasing number of studies have revealed that exosomal ncRNAs are selectively sorted, packaged, and delivered to cells and exert a vital functions in modulating various hallmarks of BCa, including proliferation, EMT process, apoptosis, lymphangiogenesis, treatment resistance, and TME remodeling. Exosomal ncRNAs are also enriched in the urine and plasma, making them promising, convenient, and effective biomarkers for BCa diagnosis and monitoring. Bioactive molecules in exosomes can become potential therapeutic targets, and exosomes can also be used as a tool for carrying therapeutic agents, therefore exosome-based BCa therapies have emerged as a hopeful approach to the currently unsatisfactory BCa therapies.

Liquid biopsy, including the detection of urine and blood-derived exosomes, has obvious unique advantages. First, samples are easy to obtain. Compared with tissue biopsy, liquid biopsy can obtain specimens conveniently and quickly, which brings less trauma and lower expenditure to patients. Second, liquid biopsy makes it easier to ensure the standardization and accuracy of operating procedures, making the test results more reproducible. Third, due to its small or noninvasive nature, liquid biopsies can be used for tumor monitoring in addition to diagnosis, which is unmatched in tissue biopsy. Since different exosomal ncRNAs present different levels of enrichment in the urine and blood, it is necessary to selectively sample urine or blood for detection based on the target exosomal ncRNAs, and often a combined detection approach is needed to improve accuracy. However, for urinary system tumors, current studies have shown that the exploration of exosomal ncRNAs may be used as biomarkers, which indicates that exosomal ncRNAs in the urine present higher medical value in the diagnosis and tumor surveillance. In contrast, in nonurinary system tumors, blood-derived exosomal ncRNAs may be a better choice.

Although in some cancers, exosome-based treatments have been applied clinically, and research on exosomes in BCa is also advancing rapidly, further research is required before clinical applications of exosome-based treatments in BCa can become a reality. The first challenge is to identify and isolate tumor-specific exosomes. Many techniques like serial centrifugation, size-based methods, and Polymer-based precipitation methods are commonly used, but none can perfectly provide us with pure and intact exosomes in an economical, convenient, and efficient way. Furthermore, how to load the bioactive substances into exosomes is also an urgent question to be solved. Improper loading processes or extreme loading may result in poor loading efficiency, unstable loading, or even complete failure. Third, although exosomal inclusions are selectively sorted and packaged, enormous inclusions still have not been identified, and how these sorting functions work as well as where these exosomes are distributed remain largely unknown. Finally, intricate interactions between exosomal bioactive molecules and their recipient cells pose a huge challenge for researchers, exosomal quality control must be settled before any clinical applications. Nonetheless, we believe that continued efforts will solve the current difficulties will eventually allow exosomes to be developed as next-generation BCa therapies.

## Author Contributions

KT, YT, XL, and ZC designed the study. YT, XL, YH, and TY collected data. ZY, ZC, DX, HL, EP, HX, and XY analyzed the data. YT and XL made the figures and tables. KT, YT, and XL drafted the article. All authors contributed to the article and approved the submitted version.

## Funding

This project was supported by the National Natural Science Foundation of China (No. 81900645), 2019 Wuhan Yellow Crane Talent Program (Outstanding Young Talents), and the Tongji Hospital (HUST) Foundation for Excellent Young Scientist (No. 2020YQ15).

## Conflict of Interest

The authors declare that the research was conducted in the absence of any commercial or financial relationships that could be construed as a potential conflict of interest.

## Publisher’s Note

All claims expressed in this article are solely those of the authors and do not necessarily represent those of their affiliated organizations, or those of the publisher, the editors and the reviewers. Any product that may be evaluated in this article, or claim that may be made by its manufacturer, is not guaranteed or endorsed by the publisher.

## Glossary

BCa: bladder cancer
EV: extracellular vesicle
ncRNAs: noncoding RNAs
miRNAs: microRNAs
lncRNAs: long noncoding RNAs
circRNAs: circular RNAs
EMT: epithelial-mesenchymal transitions
NMIBC: nonmuscle-invasive bladder cancer
MIBC: muscle-invasive bladder cancer
TURBT: transurethral resection of bladder tumor
MVB: multivesicular bodies
ILVs: intraluminal vesicles
ESCRT: endosome -sorting complexes
ALIX: apoptosis-linked gene 2-interacting protein X
piRNAs: PIWI-interacting RNAs
tsRNAs: tRNA-derived small RNAs
RISC: RNA-induced silencing complex
TME: tumor microenvironment
mRNAs: messenger RNAs
ERF: Ets2-repressor factor
UBIAD1: UbiA prenyltransferase domain containing protein 1
MET: mesenchymal to epithelial transition
PTEN: phosphatase and tensin homolog
EMT-TFs: epithelial-mesenchymal transitions related transcription factors
UCB: urothelial carcinoma of the bladder
FZD8: Frizzled-8
DUSP1: dual-specificity protein phosphatase 1
Esm1: Esm1 modulates
hWJMSCs: human umbilical cord Wharton jelly mesenchymal stem cell
CCNJ: cyclin J
VEGF: vascular endothelial growth factor
PTX: paclitaxel
DOX: doxorubicin
CAFs: cancer-associated fibroblasts
LNM: lymph node metastasis
AUC: area under the curve
CSM: cancer-specific mortality survival
RFS: recurrence-free survival
OS: overall survival
DFS: disease-free survival
HR: hazard ratio
siRNA: small interfering RNA
